# Expression of hemerythrin-like genes from the obligate aerobe *Myxococcus xanthus* improves the growth of the industrially relevant *Gluconobacter oxydans*

**DOI:** 10.3389/fmicb.2025.1734440

**Published:** 2025-12-16

**Authors:** Alexa L. Fleegal, Mason J. Stenzel, Paul Schweiger, Daniel J. Bretl

**Affiliations:** Department of Microbiology, University of Wisconsin, La Crosse, WI, United States

**Keywords:** hemerythrin, Myxococcus, Gluconobacter, oxygen-dependent, growth conditions

## Abstract

*Myxococcus xanthus* is a ubiquitous, obligately aerobic soil bacterium. *M. xanthus* has many two-component systems that serve to regulate responses to environmental stimuli. One of these systems, the multicomponent signaling system named the NmpRSTU pathway has been demonstrated to regulate genes that are predicted to be important for oxygen utilization, including the gene *mxan_5531,* which encodes a predicted hemerythrin-like protein. The family of proteins that includes hemerythrin-like proteins binds oxygen coordinated by a di-iron cofactor. Based on the sequence alignments and predictive structural analysis, we have determined that *M. xanthus* encodes at least five other predicted hemerythrin-like proteins. Of these six proteins predicted to be hemerythrin-like proteins, four were biochemically confirmed to bind oxygen *in vitro* using UV-spectroscopy. Currently, little is known about *M. xanthus* oxygen-dependent phenotypes, and the role of these hemerythrin-like proteins in *M. xanthus* is undescribed. To assess possible *in vivo* function, we chose to examine the impact of heterologous expression in the industrially relevant bacterium *Gluconobacter oxydans.* This bacterial species is used industrially to oxidize sugars to produce vinegar, vitamin C, the anti-diabetic drug miglitol, and several other products. However, due to its high oxygen demand, *G. oxydans* has a relatively slow growth rate under industrial-scale conditions. The expression of five of the hemerythrin-like proteins in *G. oxydans* led to faster doubling times and increased cell densities.

## Introduction

*Myxococcus xanthus* is a ubiquitous soil bacterium known for its complex social behaviors that include motility, microbial predation, fruiting body development, and sporulation (Bretl and Kirby, 2016; Kroos et al., 2025). Notably, *M. xanthus* is widely regarded as an obligate aerobe, yet soil is highly dynamic and the level of soil aeration is dependent on numerous factors including depth, texture, and moisture content (Du et al., 2023; Shahzad et al., 2019). Therefore, *M. xanthus* must have mechanisms to respond to changing environmental oxygen concentrations. Recently, a *M. xanthus* multicomponent signaling system named the NmpRSTU pathway has been shown to regulate genes associated with oxygen utilization in this species (Bretl et al., 2018; McAllister et al., 2025). The master sensor kinase of this system, NmpU, contains a protoglobin domain that coordinates the binding of a heme synthetic group required for oxygen binding and auto-phosphorylation activity (McAllister et al., 2025; Kitanishi et al., 2011; Fojtikova et al., 2015). NmpU subsequently coordinates a multistep, hierarchical phosphotransfer pathway culminating in the activation of the response regulator NmpR (Bretl et al., 2018; McAllister et al., 2025) that may be important for *M. xanthus* adaptation to low oxygen environments and its complex social behaviors.

NmpR has been shown to bind to and regulate multiple promoters/operons, including binding to the promoter of a putative two-gene operon consisting of the genes *mxan_5532* and *mxan_5531* (McAllister et al., 2025). The *mxan_5531* gene encodes a predicted, but uncharacterized, hemerythrin-like protein. Biologically, hemerythrins are oxygen-binding proteins that have long been described in invertebrates. Contrary to what the name of this protein family suggests, hemerythrins do not contain heme groups. Rather, oxygen binding is facilitated by two iron atoms positioned in the middle of four parallel alpha helices and a conserved set of amino acid residues (Weber and Salemme, 1980). Hemerythrins are typically octamers composed of stacked tetramers with a stacked square or “square doughnut” shape, but can also be monomeric, trimeric, or tetrameric (Klippenstein, 1980). Hemerythrin-like proteins have also been described in other eukaryotes like fungi and plants, as well as many different species of bacteria, which maintain the overall structure, but utilize an alternative iron-binding motif compared to originally described hemerythrins (Bailly et al., 2008; French et al., 2008; Alvarez-Carreño et al., 2016, 2018). Because hemerythrin-like proteins bind oxygen reversibly, there are three common states of the protein: deoxygenated, oxygenated, and autoxidized (Kao et al., 2008). The iron atoms in deoxy-hemerythrin are ferrous (Fe^2+^), while the iron atoms in oxy- and autoxidized hemerythrin are oxidized to the ferric state (Fe^3+^) (Kao et al., 2008). The deoxygenated and oxygenated states can be distinguished through spectroscopy. Specifically, an oxygenated protein has a characteristic peak of absorbance between wavelengths 330–370 nm compared to the deoxygenated state of the protein, where this peak is lost (Kao et al., 2008; Clay et al., 2020; Li et al., 2014; Ma et al., 2020; Okamoto et al., 2013; Justino et al., 2006).

Currently, there is little known about oxygen-dependent phenotypes of *M. xanthus* and the role of these hemerythrin-like proteins in *M. xanthus* physiology and behavior is unknown. An alternative approach to assessing the *in vivo* function of these proteins is the heterologous expression in another obligate aerobe with well-described growth characteristics. In this study, we expressed the *M. xanthus* hemerythrin-like genes in an industrially relevant bacterium, *Gluconobacter oxydans*. *G. oxydans* is used industrially to oxidize sugars to produce vinegar, vitamin C, the anti-diabetic drug miglitol, tanning agents dihydroxyacetone and erythrulose, and several other products (Prust et al., 2005; Deppenmeier and Ehrenreich, 2009; Qin et al., 2022; da Silva et al., 2022). However, production of these compounds is limited by the availability of dissolved O_2_ (Wehrs et al., 2019; Castan et al., 2002). Furthermore, O_2_-dependence is critically linked to the unique metabolism and physiology of *G. oxydans*. Industrial products are produced by periplasmically oriented membrane-bound dehydrogenases that oxidize their substrates outside the cell. The electrons from these oxidations are shuttled directly into the aerobic respiratory chain (Deppenmeier and Ehrenreich, 2009). Consequently, the concentration of dissolved O_2_ is rate-limiting for production yields with low dissolved O_2_ leading to slower growth, lower production yields, and higher by-product accumulation (Oosterhuis et al., 1985; Zhou et al., 2017). Continuous supply of O_2_ is an engineering challenge, and may be better addressed by altering the physiology of the bacteria. Collectively, in this study we demonstrate that *M. xanthus* encodes at least four functional hemerythrin-like proteins, which, when expressed in *G. oxydans*, result in growth improvement that may have significant industrial applications.

## Materials and methods

### Bacterial strains and plasmid construction

*Escherichia coli* strains (Supplementary Table S1) were routinely grown at 37 °C in lysogeny broth (LB, 10 g/L tryptone, 5 g/L yeast extract, 10 g/L NaCL) (Becton-Dickinson, Franklin Lakes, NJ, USA) with 250 rpm shaking. *G. oxydans* was routinely grown in yeast mannitol broth (YM, 20 g/L mannitol and 6 g/L yeast extract) at 26 °C with 250 rpm shaking. The hemerythrin genes were amplified from *M. xanthus* DZ2 (Aramayo and Nan, 2022), which was grown in Casitone Yeast Extract (Bretscher and Kaiser, 1978) at 32 °C with shaking at 220 rpm. Agar was added to 1.5% when making solid media. When necessary, kanamycin was added to the media at a final concentration of 50 μg/mL for plasmid maintenance. All plasmids (Supplementary Table S2) were purified using a high-speed plasmid mini kit (IBI Scientific, Dubuque, IA). Purity and concentration of the plasmid DNA were determined by spectrometry (Nanodrop, Thermo Scientific, Waltham, MA). PCR was performed using FailSafe polymerase with Buffer K (Epicentre Technologies, Madison, WI, USA), with all primers supplied by Eurofins Genomics (Supplementary Table S3) (Louisville, KY, USA). Template DNA for gene amplification was purified from *M. xanthus* DZ2 with a phase extraction protocol, as previously described (Bretl et al., 2018). A PCR cleanup kit (IBI Scientific, Dubuque, IA) was used to purify the PCR products. Products were subsequently cloned by restriction digestion and ligation into pET28a(+) and the commonly used plasmid for expression in acetic acid bacteria, pBBR1p452 (Supplementary Table S2) (Kallnik et al., 2010). Plasmid constructs were verified by Sanger Sequencing (Eurofins Genomics, Louisville, KY, USA). The Q5 Site-Directed Mutagenesis Kit (New England Biolabs, Ipswich, MA, USA) was used to introduce mutations to plasmids at sites that were structurally predicted to disrupt diiron binding based on comparison to characterized hemerythrins and known mutants that disrupted diiron binding (Nobre et al., 2015). Plasmids were moved to *G. oxydans* by conjugation with *E. coli* S17-1 as previously described (Kiefler et al., 2017).

### Predictive modeling and sequence alignments

The *M. xanthus* genome was searched for hemerythrin-like proteins using the blastp suite at NCBI (Altschul et al., 1997) and the Microbial Signal Transduction Database (Gumerov et al., 2023). Gene annotation is based on the numbering system from the well-annotated *M. xanthus* DK1622 strain (Goldman et al., 2006). Structure prediction was achieved with AlphaFold3 (Jumper et al., 2021), and subsequent visualization was performed with UCSF ChimeraX (Meng et al., 2023). Multiple sequence alignment of hemerythrin-like proteins was performed using Clustal Omega (Madeira et al., 2024). The generated alignment was adjusted using Jalview (v2.0) (Waterhouse et al., 2009), specifically to move a sequence gap at position 58 to position 73. Alignments were annotated using ESPript (v.3.0) (Robert and Gouet, 2014) and percent protein identities were determined using BLASTp (Altschul et al., 1997).

### Protein expression and purification

Sequence-confirmed recombinant pET28a plasmids expressing the *M. xanthus* hemerythrin-like genes were transformed into chemically competent *E. coli* BL21 (DE3) by heat shock. Successful transformants were grown in Terrific Broth (12 g/L tryptone, 24 g/L yeast extract, 5.04 g/L glycerol, 17 mM KH_2_PO_4_, 72 mM K_2_HPO_4_) at 37 °C and 250 rpm until OD_600_ = 0.8 was reached, then 1.0 mM isopropyl thiogalactoside (IPTG) was added, followed by overnight incubation at 22 °C and 250 rpm. Cells were harvested by centrifugation, and the pellets were suspended in wash buffer (50 mM sodium phosphate, 300 mM NaCl, 0 mM imidazole, pH 8.0). Cells were either lysed by the addition of 2.4 g of CelLytic powder (Sigma-Aldrich) per 1 L of original culture and rocking at room temperature for 1.5 h or by an in-house lysis method. The in-house lysis buffer was composed of 1 mg/mL lysozyme, 0.1% Tween 20, and 0.1% Triton X-100. Pellets were subjected to two freeze–thaw cycles at −80 °C and 37 °C, then 0.05 mg/mL DNase I and 10 mM MgCl_2_ were added, followed by rocking at room temperature for 1 h. Lysates were centrifuged at 15,000 x g for 30 min at 4 °C. Poly-Prep Chromatography Columns (Bio-Rad Laboratories) were prepared by adding 2 mL of His-Select Cobalt Affinity Gel (Sigma-Aldrich) and equilibrated with deionized H_2_O and 0 mM imidazole wash buffer. The supernatants were added to the columns, then the columns were washed with an increasing gradient of 0 to 250 mM imidazole. Whole fractions were collected and checked for purity via SDS-PAGE. Chosen fractions were dialyzed overnight at 4 °C in 1 L of dialysis buffer composed of 50% glycerol, 25 mM Tris pH 8.0, and 175 mM NaCl. Protein concentrations were determined via Bradford Assay using bovine serum albumin as a standard (Bradford, 1976).

### Biochemical characterization of hemerythrin-like proteins

Isolated hemerythrin-like proteins were characterized by UV–Vis spectrometry in 200 μL aliquots in a 96-well microtiter plate. Absorbance spectra between 300 and 600 nm were recorded immediately after dialysis using pre-dialysis buffer as a blank. Deoxygenation was achieved with the addition of 40x molar excess sodium dithionite and 18 h incubation in an anaerobic glove chamber (85% nitrogen, 10% hydrogen, 5% carbon dioxide atmosphere) (Plas-Labs, Controlled Atmosphere Chamber 855-AC). The wells were sealed with Parafilm M (Amcor plc, Zürick, CH) to prevent overnight volume loss. Deoxygenated spectra were recorded immediately after mixing the wells and removal from the anaerobic glove chamber.

### Growth curves of *Gluconobacter oxydans*

Individual overnight cultures of *G. oxydans* containing an expression plasmid or the empty vector control were diluted to ~0.1 OD_600_ in YM with kanamycin and 400 μL of culture was distributed onto a 48-well plate (Corning Incorporated, Corning, NY, USA). Growth was monitored using a SpectraMax M3 plate reader (Molecular Devices, San Jose, CA, USA) at 600 nm and 26 °C with continuous shaking between readings. Growth curves were constructed in RStudio using data from at least two biological replicates and four technical replicates. Generation time, carrying capacity, and area under the logistic curve were calculated using the Growthcurver package and the (Sprouffske and Wagner, 2016). Lag time was calculated using the Microbial Lag Calculator fit to the logistic model (Smug et al., 2024). All statistics were done in R using the Kruskal-Wallis test followed by a post-hoc Wilcoxon rank sum with the Benjamini-Hochberg correction for multiple comparisons.

## Results

### *Myxococcus xanthus* hemerythrin-like proteins share conserved amino acid residues and predicted structure with *Escherichia coli* YtfE

**Figure 1:**
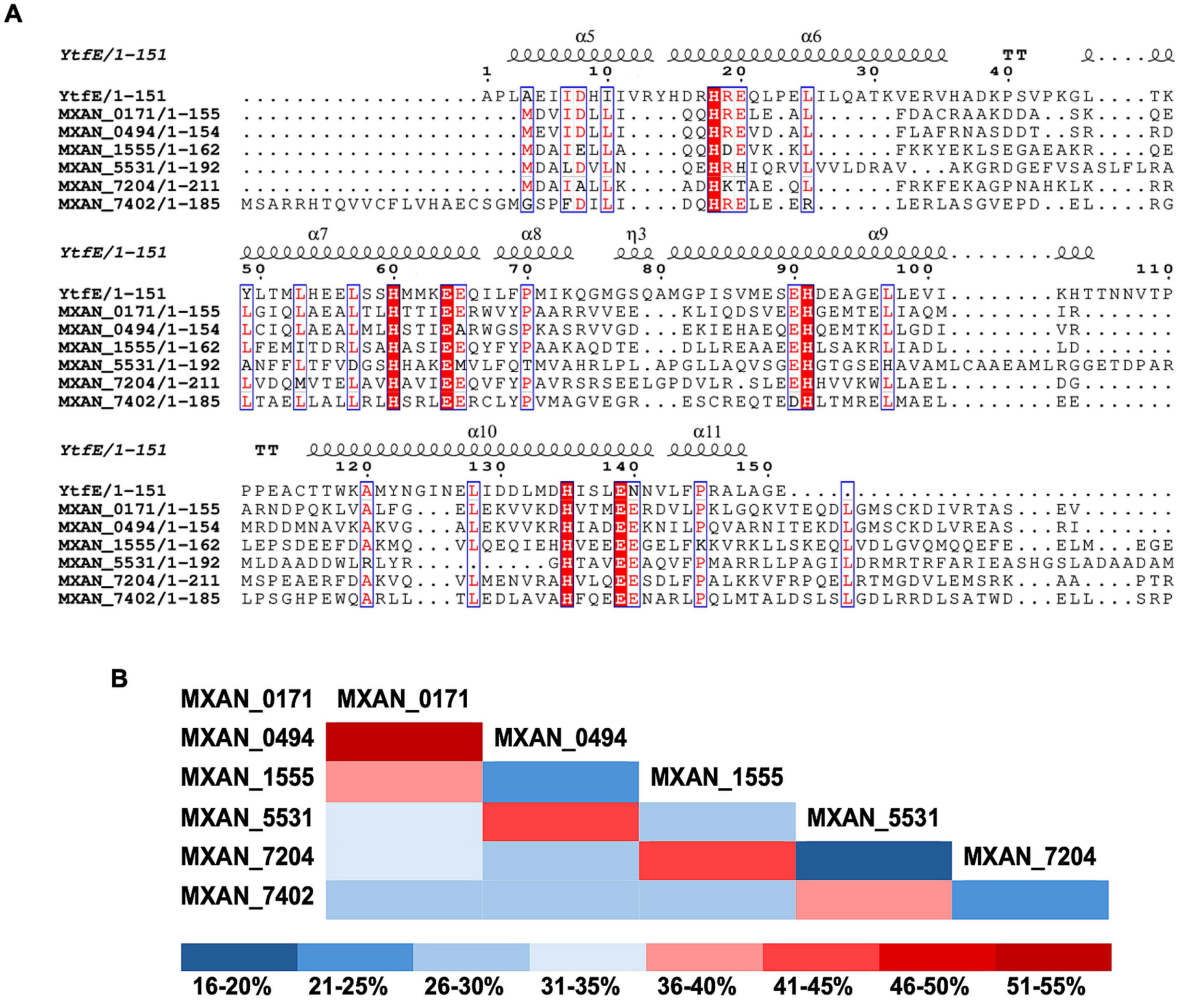
Sequence alignment of *Myxococcus xanthus* predicted hemerythrin-like proteins suggests a conserved iron-binding motif. **(A)** Sequence alignment of *Escherichia coli* YtfE hemerythrin-like protein (PDB ID: 7BHA) and *Myxococcus xanthus* hemerythrin-like proteins. Sequences are trimmed to depict only the relevant hemerythrin-like domains. The YtfE alpha helices are depicted above the sequence alignment, and the amino acid number is also based on YtfE. Alignment was annotated in ESPript (version 3.0) (Robert and Gouet, 2014). Red boxes indicate residues that are strictly conserved, and red letters indicate high sequence similarity. **(B)** Heat map indicating percent identity shared between *Myxococcus xanthus* hemerythrin-like proteins. Percent protein identities were determined using BLASTp. Percent identities range from 20 to 54.97%.

**Figure 2:**
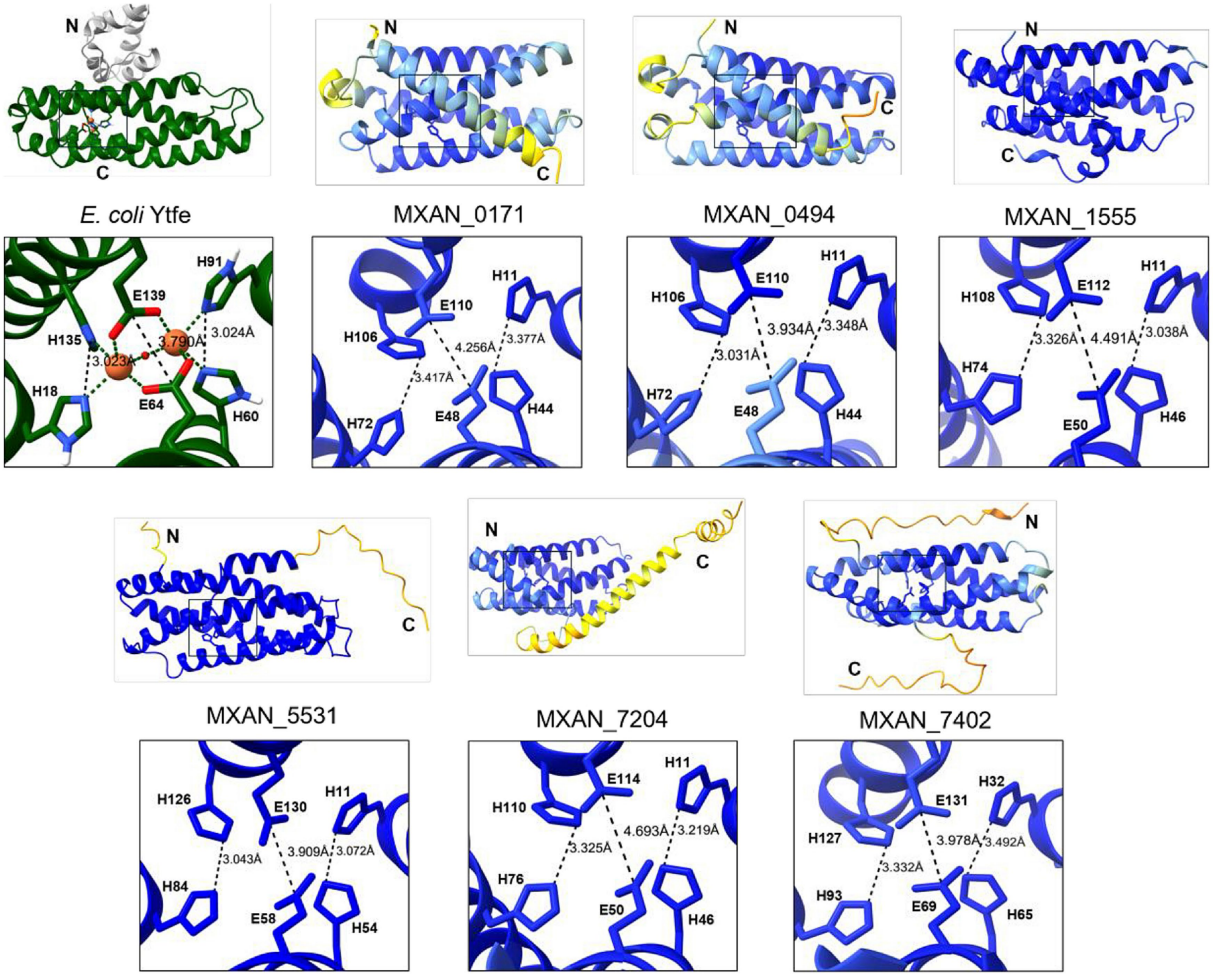
Comparison of the YtfE crystal structure (PDB ID: 7BHA) and *Myxococcus xanthus* hemerythrin-like protein structure predictions indicates similarities. The hemerythrin-like domain of YtfE is colored green. Indicated in the insets are the residues that align in sequence with the iron-binding residues of YtfE and distances between residues. The YtfE inset depicts bound iron (orange spheres) and oxygen (red sphere) atoms. Structure predictions were performed with AlphaFold3. Residues with very high model confidence are dark blue and residues with very low confidence are orange. Visualization was achieved with UCSF ChimeraX.

In addition to *mxan_5531*, we identified at least five other predicted hemerythrin-like genes in the *M. xanthus* genome: *mxan_0171*, *mxan_0494*, *mxan_1555*, *mxan_7204*, and *mxan_7402*. All six of the predicted proteins encoded by these genes are single-domain hemerythrin-like proteins ~150–200 amino acids in length. The *E. coli* hemerythrin-like domain-containing protein, YtfE (RCSB: 7BHA), was chosen as a model for comparison of the predicted *M. xanthus* hemerythrin-like proteins because YtfE has an available crystal structure and an iron-binding region consisting of two glutamate and four histidine residues. YtfE is a multi-domain protein, with an N-terminal ScdA_N domain of unknown function (Alvarez-Carreño et al., 2018; Silva et al., 2021; Lo et al., 2016). A multiple-sequence alignment of the hemerythrin-like domain of YtfE and the six single-domain *M. xanthus* hemerythrin-like proteins (MXAN_0171, MXAN_0494, MXN_1555, MXAN_5531, MXAN_7204, and MXAN_7402) demonstrated all six *M. xanthus* hemerythrin-like proteins have the conserved H-HxxxE-H-HxxxE iron-binding motif (Figure 1). When comparing the *M. xanthus* proteins against themselves, the percent identity between each predicted *M. xanthus* hemerythrin-like protein suggests that some of these genes may have been the result of gene duplication, while other genes are more distantly related. Percent identities ranged from only 20% between MXAN_5531 and MXAN_7204, to 54.97% identity between the most similar pair, MXAN_0171 and MXAN_0494 (Figure 1B). All six *M. xanthus* hemerythrin-like proteins have a predicted structure of four parallel alpha helices with an overall left-handed path, and the aforementioned histidines and glutamates that are positioned on the inside of the helices. The side chains of these residues point toward each other, and the distances between them are comparable to those of the iron-binding pocket of YtfE (Figure 2). This initial comparison of amino acid sequence and predicted structure highly suggested these *M. xanthus* proteins are hemerythrin-like proteins.

### *Myxococcus xanthus* MXAN_0171, MXAN_1555, MXAN_5531, and MXAN_7204 having characteristic absorbance spectra of hemerythrin-like proteins

**Figure 3:**
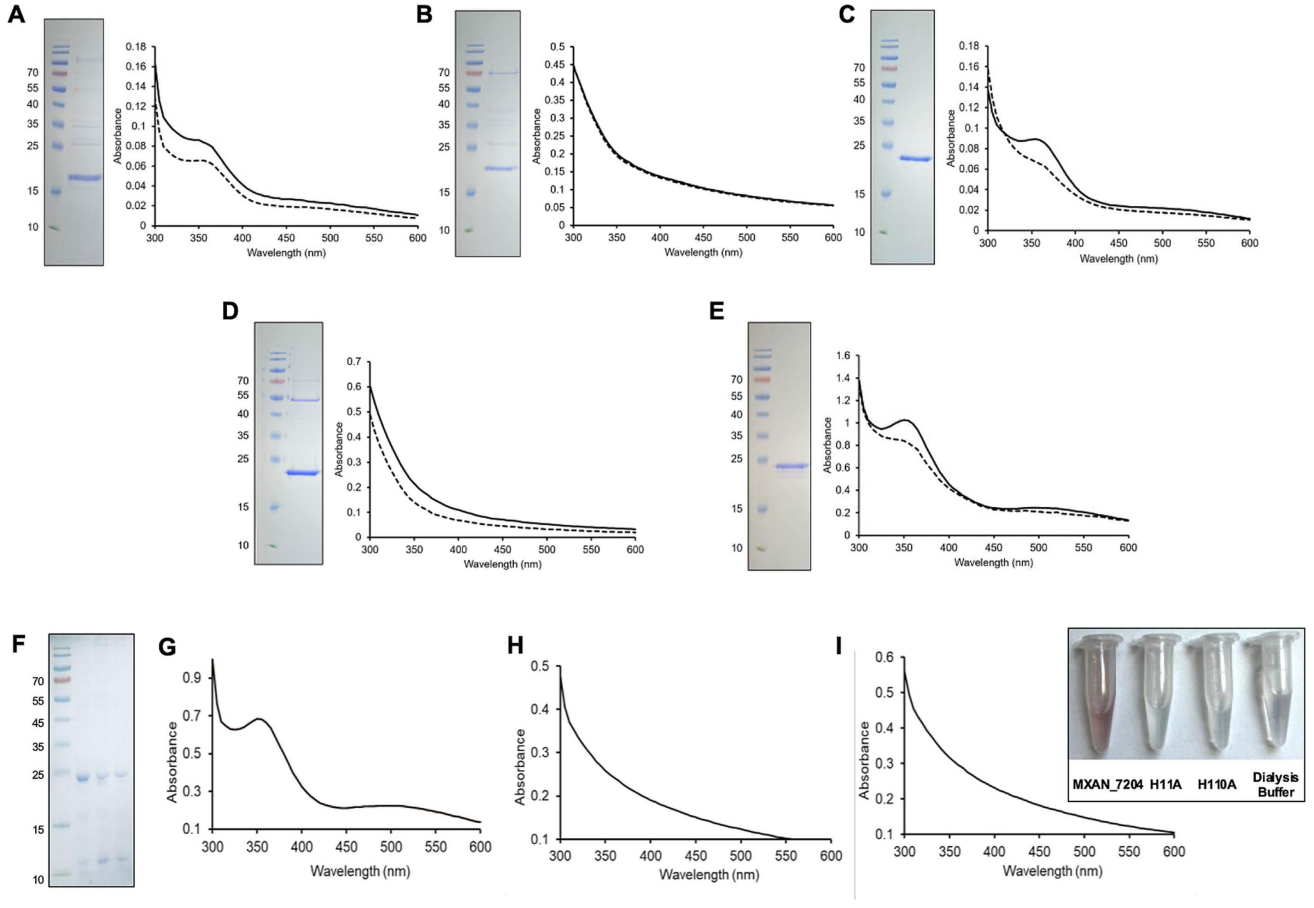
*Myxococcus xanthus* encodes at least four functional hemerythrin-like proteins. **(A–E)** SDS-PAGE and absorbance spectra of as-isolated and deoxygenated for **(A)** MXAN_0171, **(B)** MXAN_0494, **(C)** MXAN_1555, **(D)** MXAN_5531, **(E)** MXAN_7204. The as-isolated spectra (solid lines) were recorded immediately after purification; the post-dithionite spectra (dashed lines) were recorded after overnight treatment with 40x molar excess sodium dithionite in an anaerobic chamber. The decrease in absorbance across the relevant 330–370 nm range in all proteins except for MXAN_0494 is indicative of deoxygenation. Molecular-weight ladders are labeled (kDa), expected product sizes are as follows: MXAN_0171: 19.2 kDa, MXAN_0494: 19.1 kDa, MXAN_1555: 20.3 kDa, MXAN_5531: 23.2 kDa, MXAN_7204: 24.9 kDa. **(F)** SDS_PAGE of purified MXAN_7204, H11A and H110A variants, left to right. **(G–I)** As-isolated absorbance spectra for MXAN_7204 **(G)** and its variants MXAN_7204 H11A **(H)**, and MXAN_7204 H110A **(I)**. The inset shows the color of MXAN_7204 after purification compared to the two variants and dialysis buffer.

The standard method to demonstrate oxygen binding by hemerythrin-like proteins is to record the oxygenated spectrum, then anaerobically reduce the proteins with the addition of a strong reducing agent, such as sodium dithionite. Iron atoms within the binding pocket that are successfully reduced to the ferrous state, will result in a deoxygenated protein and the resulting absorbance spectrum will be lower than the as-isolated spectrum from 330–370 nm (Kao et al., 2008; Clay et al., 2020; Li et al., 2014; Ma et al., 2020; Okamoto et al., 2013; Justino et al., 2006). So, to address whether the predicted *M. xanthus* proteins are functional hemerythrin-like proteins, we sought to purify and assess the absorbance spectra of each. First, protein purification of the *M. xanthus* hemerythrin-like proteins was done empirically. Initially, all the protein expression constructs were designed with N-terminal 6xHis-tags. Only purification of MXAN_5531 resulted in a single, prominent band of the expected size, suggesting the N-terminal 6xHis tag may interfere with proper protein production and/or folding. Therefore, cloning was repeated to express each protein with a C-terminal 6xHis tag for all but MXAN_5531. Subsequent purification resulted in a single prominent band of the expected size for all proteins besides MXAN_7402. Despite several attempts, we were never able to purify MXAN_7402. Of the five proteins that successfully purified, it was notable that MXAN_0171, MXAN_1555, and MXAN_7204 were each pink in solution (data not shown and Figure 3I) and had a peak of absorbance at ~350 nm, both well-described characteristics of hemerythrins and related proteins (Kao et al., 2008; Clay et al., 2020; Justino et al., 2006; Okamoto et al., 2013; Ma et al., 2020) (Figure 3). To deoxygenate the hemerythrin-like proteins, sodium dithionite was added at 40x molar excess in an anaerobic chamber (Ma et al., 2020; Clay et al., 2020; Li et al., 2014; Okamoto et al., 2013; Justino et al., 2006; Hamada et al., 1962). Overall, four of them were deoxygenated after the addition of sodium dithionite: MXAN_0171, MXAN_1555, MXAN_5531, and MXAN_7204 (Figure 3), as demonstrated by the loss of absorbance between wavelengths 330–370 nm. These spectra data in addition to the sequence similarity to the YtfE hemerythrin-like domain also supports the presence of an oxo-bridged diiron(III) site in the hemerythrin-like proteins from *M. xanthus* and is consistent with oxygen binding seen in other characterized hemerythrins. The as-isolated spectrum and post-dithionite spectrum of MXAN_0494 were unchanged, suggesting that MXAN_0494 may not bind iron and/or oxygen under the *in vitro* conditions tested.

Finally, as a model for the importance of the amino acids within the iron binding pocket (Figures 1, 2), the *mxan_7204* expression vector was mutated resulting in two independent MXAN_7204 variants, H11A and H110A. Following purification of these variants, as-isolated absorbance spectra were collected. These spectra notably lacked the characteristic absorbance peak between 330–370 nm and were no longer visibly pink, indicating these histidines are each necessary to coordinate binding of the irons and therefore oxygen binding (Figures 3F–I).

### Expression of *Myxococcus xanthus* hemerythrin-like genes in *Gluconobacter oxydans* results in improved growth

**Figure 4:**
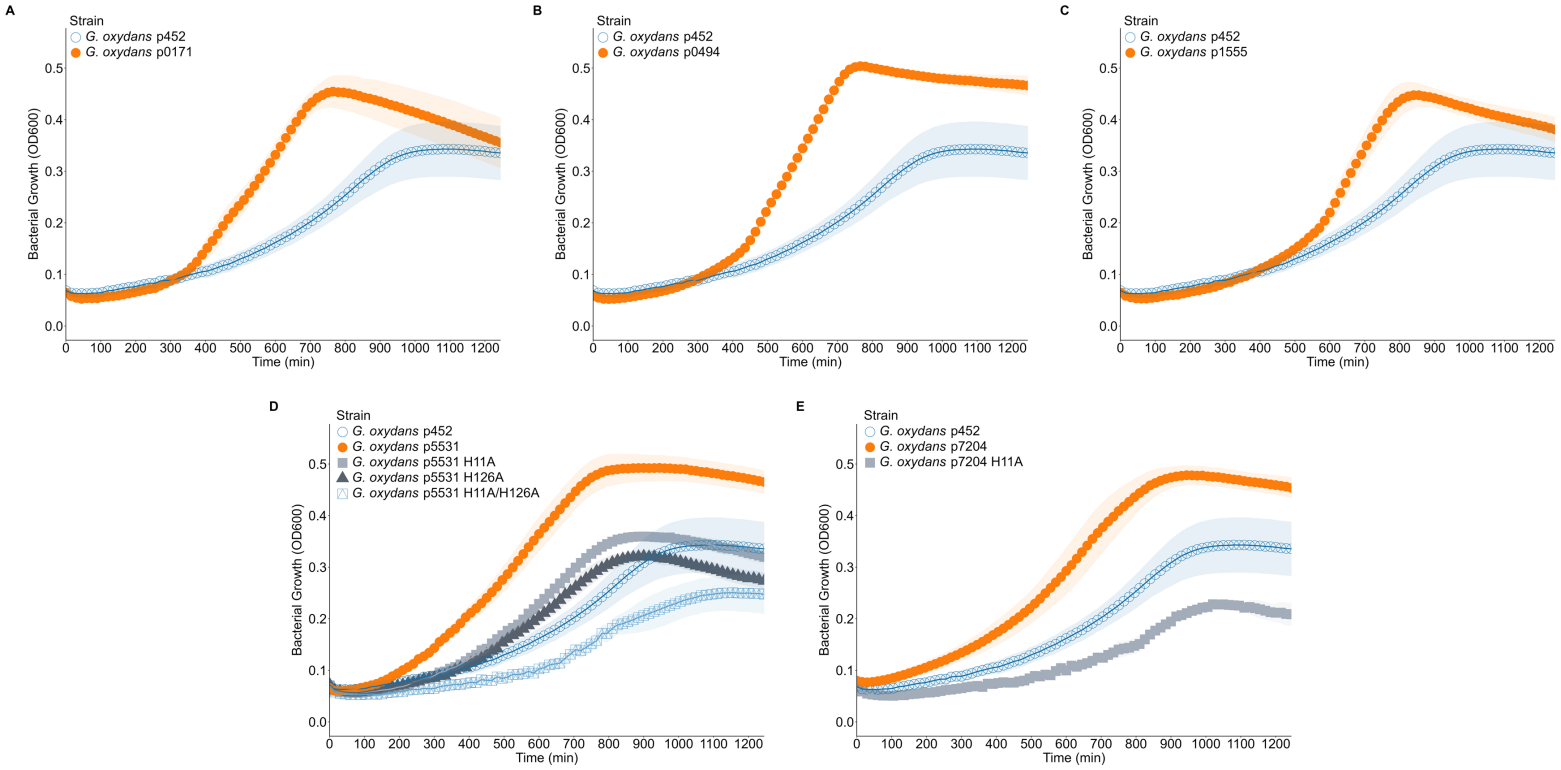
Expression of an *Myxococcus xanthus* predicted hemerythrin-like protein in *Gluconobacter oxydans* results in improved growth compared to *Gluconobacter oxydans* carrying the overexpression vector, while mutation of the MXAN_5531 and MXAN_7204 iron-binding motifs results in decreased growth benefits. **(A–E)** Growth curves of *Gluconobacter oxydans* 621H expressing a *Myxococcus xanthus* hemerythrin-like protein MXAN_0171 **(A)**, MXAN_0494 **(B)**, MXAN_1555 **(C)**, MXAN_5531 **(D)**, and MXAN_7204 **(E)**. Wild-type *Gluconobacter oxydans* 621H containing the empty vector pBBR1p452 is included as comparison for the improved growth. Relevant oxygen binding-deficient hemerythrins are shown in **(D,E)**. 95% confidence intervals are plotted as ribbons.

Having established that at least four of *M. xanthus* predicted hemerythrin-like proteins bind oxygen *in vitro,* we sought to determine if they were also functional *in vivo.* However, very little is known about oxygen-dependent phenotypes in *M. xanthus,* complicating studies to examine functionality in this species. Therefore, we chose to examine the *in vivo* function of the hemerythrin-like proteins in the industrially relevant bacterium *G. oxydans* as a possible method for improving oxygen utilization by this obligately aerobic organism. *G. oxydans* strains expressing the *M. xanthus* hemerythrin-like genes were grown in a low volume, 48-well plate assay, which has reduced aeration compared to growth in flasks (Running and Bansal, 2016). All hemerythrin expressing strains reached higher final cell densities than the strain containing the empty vector (Figure 4). Higher final cell densities also reflected the higher carrying capacities for hemerythrin expressing strains (all having *p* < 0.05) (Table 1). All hemerythrin expression strains also had faster generation times compared to the empty vector-containing strain (all having *p* < 0.001) (Table 1). Interestingly, strains *G. oxydans* p5531 (*p* < 0.001) and *G. oxydans* p7204 (*p* = 0.028) also had significantly shorter lag times than the *G. oxydans* p452 empty vector strain (Table 1). The area under the curve (AUC) summarizes growth by integrating carrying capacity, growth rate, and initial population size into a single value (Sprouffske and Wagner, 2016). We observed higher AUC values for all hemerythrin expression strains (all having *p* < 0.001) (Table 1). Taken together, these data suggest that increased capacity for oxygen-binding in low aeration conditions improved growth of *G. oxydans*. Interestingly, although the *in vitro* absorbance spectrum suggested MXAN_0494 does not bind O_2_ *in vitro* (Figure 3), these data suggest that it nonetheless has a potential advantageous function *in vivo*.

**Table 1:** Growth characteristics^a^ of *Gluconobacter oxydans* expressing hemerythrin-like proteins.

Strain	Generation time (min)	Carrying capacity	Area under curve (AUC)	Lag time (min)
p452	112 ± 4^b^	0.308 ± 0.025	168.6 ± 11.1	360 ± 20
p0171	49^***^ ± 2^c^	0.367^*^ ± 0.020	275.0^***^ ± 10.9	324 ± 13
p0494	55^***^ ± 3	0.447^***^ ± 0.006	312.1^***^ ± 3.9	328 ± 14
p1555	57^***^ ± 2	0.371^*^ ± 0.010	241.8^***^ ± 8.2	400 ± 11
p5531	79^***^ ± 4	0.440^***^ ± 0.011	329.0^***^ ± 9.9	160^***^ ± 8
p5531 H11A	72^***^ ± 3	0.297 ± 0.005	206.6^**^ ± 3.3	326 ± 13
p5531 H126A	70^***^ ± 3	0.252 ± 0.007	175.1 ± 5.7	340 ± 11
p5531 H11A/H126A	108 ± 6	0.170^**^ ± 0.001	84.9^**^ ± 2.0	518^***^ ± 27
p7204	85^***^ ± 4	0.412^**^ ± 0.011	274.6^***^ ± 8.9	193^**^ ± 6
p7204 H11A	101 ± 9	0.185^**^ ± 0.001	94.6^***^ ± 5.1	486^***^ ± 32

^a^Generation time, carrying capacity, and AUC calculated using the R package Growthcurver (Sprouffske and Wagner, 2016). Lag time calculated using the Microbial Lag Calculator fit to the logistic model (Smug et al., 2024).

^b^mean ± SEM.

^c^*p ≤ 0.05; **p ≤ 0.01; ***p ≤ 0.001 in comparison to *Gluconobacter oxydans* p452.

To assess if the coordination of iron and subsequent oxygen binding of these hemerythrin-like proteins is necessary for the observed growth improvement, mutations were introduced to change the histidines predicted to be involved in iron binding (Figure 2). The histidines selected also correspond to mutants known to eliminate iron binding in hemerythrin domains (Nobre et al., 2015). As a proof of concept, we made these mutations in two genes: *mxan_5531* and *mxan_7204*. Expression of MXAN_5531 variants H11A and H126A resulted in a reduction of growth when compared to expression of wild-type MXAN_5531. Specifically, both variants reached similar final cell densities to the empty vector strain and had similar carrying capacities (p5531 H11A, *p* = 0.333; p5531 H126A, *p* = 0.144) (Figure 4D and Table 1). Although *G. oxydans* p5531 H11A and H126A had similar generation times to the p5531 expression stain (*p* = 0.298; *p* = 0.162), their lag times and AUC values appeared more similar to the *G. oxydans* p452 empty vector strain. The shift in the growth curve (Figure 4D) and growth characteristics (Table 1) both appearing more similar to *G. oxydans* p452 suggest these mutations lead to decreased O_2_ binding. Furthermore, a variant with both mutations (p5531 H11A/H126A) resulted in slower doubling time than the p5531 expression strain (*p* = 0.002) (Table 1). This *G. oxydans* p5531 H11A/H126A strain also had a lower final density, carrying capacity (*p* = 0.032), and AUC (*p* = 0.003) while having a significantly longer lag time (*p* < 0.001) compared to wild-type *G. oxydans* expressing the empty vector (Figure 4D and Table 1). These data demonstrate the key histidine residues that make up the iron binding, and therefore oxygen binding pocket, are necessary for the growth advantage seen in the strains expressing the wild-type hemerythrins. This conclusion is further supported by the expression of a corresponding mutation in *mxan_7204*. The first histidine in the binding motif was changed to an alanine, resulting in the H11A variant. Expression of this variant resulted in a loss of oxygen binding *in vitro,* indicated by a loss of absorbance (Figure 3) and a negative impact on *G. oxydans* growth (Figure 4E). This was seen not only by lower final cell densities but lower carrying capacity (*p* = 0.034) and AUC (*p* < 0.001) but longer lag time (*p* < 0.001) compared to *G. oxydans* p452 (Table 1). This negative growth phenotype could be caused by the expression of non-functional hemerythrins or the formation of misfolded proteins aggregating into inclusion bodies that interfere with normal cell functions and increasing the metabolic burden. Importantly, these data demonstrated that the growth advantage observed when expressing the *M. xanthus* hemerythrin-like genes is dependent on their ability to bind oxygen.

## Discussion

Functions for hemerythrin proteins vary widely across the domains of life, ranging from oxygen transport in eukaryotes to highly specialized functions in aerobic and anaerobic bacteria. Examples in bacteria include oxygen delivery to methane monooxygenases in *Methylococcus capsulatus* (Chen et al., 2012), sequestration of oxygen from oxygen-sensitive enzymes in *Campylobacter jejuni* (Kendall et al., 2014), or repair of iron–sulfur clusters in pathogenic *E. coli* (Lo et al., 2016). In an organism like *M. xanthus*, which requires oxygen to grow, there are likely several different strategies for oxygen utilization, perhaps explaining the presence of multiple hemerythrin-like proteins encoded in its genome. The initial interest in these hemerythrin-like proteins was sparked by the observation that the NmpRSTU system of *M. xanthus* regulates expression of an oxygen-utilization regulon in *M. xanthus*, which includes autoregulation of the *nmpRSTU* genes, a high oxygen-affinity cytochrome c oxidase (cbb3), a heme biosynthetic gene, several stress response genes, and the hemerythrin-like protein encoding gene, *mxan_5531* (McAllister et al., 2025). We have now demonstrated that *M. xanthus* encodes multiple hemerythrin-like proteins.

To better understand the function of these hemerythrin-like genes in *M. xanthus*, we performed a sequence and structural comparison of each protein with the *E. coli* YtfE. These comparisons revealed that the six proteins from *M. xanthus* share the characteristic hemerythrin-like domain structure comprised of four parallel alpha helices with a conserved H-HxxxE-H-HxxxE iron-binding motif positioned in the center (Figures 1A, 2). Additionally, the distances between the residue side chains in each motif are predicted to be similar to that of YtfE, suggesting a high likelihood of functionality (Figure 2). Interestingly, there was a wide range of percent identities between the *M. xanthus* proteins, suggesting multiple distinct gene duplications and/or acquisitions led to this genomic arrangement (Figure 1B). Furthermore, the data presented here indicate that at least four of the six hemerythrins are functional, indicating that the conserved binding-motif is paramount in the preservation of functionality. Spectral analysis confirmed four of these hemerythrin-like proteins to be functional *in vitro* as demonstrated by a decrease in absorbance between wavelengths 330–370 nm (Figure 3). It is not clear why a loss of absorbance was not seen for MXAN_0494 when reduced with sodium dithionite. It is possible that different growth or assay conditions may optimize oxygen binding of this protein. Yet, this protein still provided a growth advantage when expressed in *G. oxydans*, suggesting it does have a function *in vivo*. One difference in the expression of MXAN_0494 in *E. coli* and *G. oxydans* was the absence of a His-tag when expressed in *G. oxydans*. This tagless expression might allow proper *in vivo* folding and/or iron incorporation. Finally, it is also unclear why MXAN_7402 failed to be purified. To date, we have tried moving the 6xHis tag from the N-term to the C-term of the protein, and cloned truncated versions of the gene to eliminate predicted unstructured domains that may interfere with solubility (Figure 2). We have also examined codon bias in *E. coli* and found only three codons that were poorly adapted. This suggests that the low occurrence of poorly adapted codons is likely not the reason purification was not successful. However, the lack of successful protein purification does not mean that MXAN_7402 is not a functional hemerythrin-like protein. For example, MXAN_7402 could also be a functional hemerythrin-like protein in *M. xanthus* but is toxic when overexpressed in *E. coli*. Further investigation into MXAN_0494 and MXAN_7402 to determine their functions will be conducted.

We investigated possible *in vivo* function of the *M. xanthus* hemerythrin-like proteins using a *G. oxydans* heterologous expression system. Oxygen availability is critically important for the metabolic activity of acetic acid bacteria, such as *G. oxydans*. Acetic acid bacteria are obligate aerobes used in the industrial production of vinegar, vitamin C, tanning agents dihydroxyacetone and erythrulose, cellulose, and levan (Raspor and Goranovic, 2008; Deppenmeier et al., 2002; Yassunaka Hata et al., 2023; da Silva et al., 2022). Most of these industrial applications exploit periplasmically-oriented membrane-bound dehydrogenases. These dehydrogenases oxidize sugars, polyols, and alcohols and shuttle electrons directly into the aerobic respiratory chain. The resulting products are released into the medium and are extracted for industrial application. Oxygen limitation decreases productivity due to impaired respiratory chain function. For example, in vinegar production, even a brief period of oxygen limitation decreased ethanol oxidation rates and acetic acid production. This is caused by a 20 and 50% reduction in activity of the alcohol and aldehyde dehydrogenases, respectively (Hitschmann and Stockinger, 1985; Zheng et al., 2018).

Oxygen limited conditions not only decrease production rates due to impaired respiratory chains but also decreases the intracellular energy charge of the cell by decreasing ATP yields (Hitschmann and Stockinger, 1985; Zheng et al., 2018). This impacts the ability of the cell to mitigate toxic metabolic byproducts. For instance, the mechanisms by which acetic acid bacteria resist the effects of toxic metabolic byproducts involves proton pumps (Matsushita et al., 2005), ABC transporters (Nakano and Fukaya, 2008), and ATP-dependent stress response proteins GrpE-DnaK-DnaJ involved in protein folding (Okamoto-Kainuma et al., 2004). These resistance mechanisms require sufficient O_2_ availability to maintain the function of the respiratory chain to generate ATP and proton motive force.

Improved oxygenation increases the activity and productivity of membrane-bound dehydrogenases of acetic acid bacteria leading to improved production of acetic acid (Zheng et al., 2018), dihydroxyacetone (Zheng et al., 2016; de la Morena et al., 2019), erythrulose (Pan et al., 2016), gluconate (Oosterhuis et al., 1985), and xylonic acid (Zhou et al., 2017), as well as improved growth (Romero et al., 1994). These improved rates are achieved by increasing aeration. The drawback of this approach is that excessive aeration can decrease yields due to volatilization of the substrates or products, such as ethanol and acetic acid (Rubio-Fernández et al., 2004). Supplying O_2_ or compressed air during production also increases energy-associated costs. Maintaining oxygen transfer rates without volatilization or additional cost of aeration is desirable for industrial productions involving acetic acid bacteria to ensure high yields and process stability. One approach to achieve this is to use oxygen-binding proteins.

We demonstrated that expression of at least five *M. xanthus* hemerythrin-like proteins resulted in higher final cell densities and decreased generation times. This phenotype is consistent with the heterologous expression of bacterial hemoglobins, which are also small, soluble, oxygen-binding proteins. For example, heterologous expression in *E. coli* of a truncated globin from *M. xanthus* resulted in higher cell densities in culture (Singh et al., 2019). Furthermore, the heterologous expression of a bacterial hemoglobin from *Vitreoscilla stercoraria* has been investigated as a method to enhance oxygen-dependent growth and microbial production yields. Heterologous expression of VHb resulted in increased cell density, shorter generation times, or both in a wide variety of bacterial and yeast species (Bhave and Chattoo, 2003; Khosla and Bailey, 1988; Pablos et al., 2011; Zhang et al., 2007) and improved production of numerous products in a variety of commercially important microbes (Mirończuk et al., 2019; Pablos et al., 2011). In *G. oxydans*, VHb expression led to a 18.60% increase in biomass in low aeration small batch cultures and an 30.37% increase in dihydroxyacetone production. Low aeration bioreactors further improved both biomass (23.13% increase) and dihydroxyacetone production (37.36% increase) (Li et al., 2010). In this study, hemerythrin expression improved biomass in *G. oxydans* 19.16–45.13% based on carrying capacity (Table 1). The large improvement in growth in *G. oxydans* expressing *M. xanthus* hemerythrins suggests that O_2_-dependent productions, such as dihydroxyacetone, may be even more dramatically improved using hemerythrins due to their O_2_-binding capacity and the increases in biomass.

Expression of hemerythrins may have advantages over globin domain proteins. Namely, hemerythrins do not require the more complex heme biosynthetic group for activity. We have successfully demonstrated that expression of *M. xanthus* hemerythrin-like protein can serve to improve the growth of *G. oxydans*. As previously stated, growth of *G. oxydans* is critically dependent on oxygen and thus oxygen availability is often a limiting factor on product yields (Oosterhuis et al., 1985; Zhou et al., 2017). Our future studies will include scaling up the growth curve analysis, O_2_ monitoring during cell growth, and monitoring production of relevant industrial molecules such as the production of dihydroxyacetone from glycerol (Deppenmeier et al., 2002).

## Data Availability

The raw data supporting the conclusions of this article will be made available by the authors, without undue reservation.
