# Supplementary material for: Expression of hemerythrin-like genes from the obligate aerobe Myxococcus xanthus improves the growth of the industrially relevant Gluconobacter oxydans
Source: Front Microbiol. 2025 Dec 16;16:1734440. doi: 10.3389/fmicb.2025.1734440 (PMC12753109; doi:10.3389/fmicb.2025.1734440)
Supplement: Supplementary file 1 [file Table_1.DOCX]

**Supplemental Table 1:** Bacterial strains used in this study

| **Strain** | **Description** | **Source** |
| --- | --- | --- |
| *E. coli* BL21 (DE3) | fhuA2 [lon] ompT gal (λ DE3) [dcm] ∆hsdS λ DE3 = λ sBamHIo ∆EcoRI-B int::(lacI::PlacUV5::T7 gene1) i21 ∆nin5 | Life Technologies |
| *E. coli* DH5α | *fhuA2 Δ(argF-lacZ)U169 phoA glnV44 Φ80 Δ(lacZ)M15 gyrA96 recA1 relA1 endA1 thi-1 hsdR17* | New England Biolabs |
| *E. coli* S17-1 | *recA pro hsdR RP4-2-Tc::Mu-Km::Tn7* integrated into the chromosome, *strR spcR tmpR*. | ATCC 47055 |
| *G. oxydans* 621H | Wildtype | DSM 2343 |
| p452 | *G. oxydans* 621H containing pBBR1p452 | This study |
| p0171 | *G. oxydans* 621H containing p452_mxan0171 | This study |
| p0494 | *G. oxydans* 621H containing p452_mxan0494 | This study |
| p1555 | *G. oxydans* 621H containing p452_mxan1555 | This study |
| p5531 | *G. oxydans* 621H containing p452_mxan5531 | This study |
| p5531 H11A | *G. oxydans* 621H containing p452_mxan5531 _H11A | This study |
| p5531 H126A | *G. oxydans* 621H containing p452_mxan5531 _H126A | This study |
| p5531 H11A/H126A | *G. oxydans* 621H containing p452_mxan5531 _H11A/H126A | This study |
| p7204 | *G. oxydans* 621H containing p452_mxan7204 | This study |
| p7204 H11A | *G. oxydans* 621H containing p452_mxan7204 _H11A | This study |

**Supplemental Table 2:** *E. coli* and *G. oxydans* expression plasmids used in this study

| **Plasmids** | **Description** | **Source** |
| --- | --- | --- |
| pBBR1p452 | pBBR1MCS-2 (Kovach et al. 1995) derivative containing the 5´-UTR of gox0452 and a C-terminal Streptag II; Kan^R^ | Kallnik et al., 2010 |
| p452_mxan0171 | pBBR1p452-ST derivative with *mxan0171* cloned into between EcoRI and HindIII sites; Kan^R^ | This study |
| p452_mxan0494 | pBBR1p452-ST derivative with *mxan0494* cloned into between EcoRI and HindIII sites; Kan^R^ | This study |
| p452_mxan1555 | pBBR1p452-ST derivative with *mxan1555* cloned into between EcoRI and HindIII sites; Kan^R^ | This study |
| p452_mxan5531 | pBBR1p452-ST derivative with *mxan5531* cloned into between EcoRI and HindIII sites; Kan^R^ | This study |
| p452_mxan5531_H11A | p452_mxan5531 derivative with H11A mutation in *mxan5531* gene; Kan^R^ | This study |
| p452_mxan5531_H126A | p452_mxan5531 derivative with H126A mutation in *mxan5531* gene; Kan^R^ | This study |
| p452_mxan5531_H11A/H126A | p452_mxan5531 derivative with the H11A and H126A mutations in *mxan5531* gene; Kan^R^ | This study |
| p452_mxan7204 | pBBR1p452-ST derivative with *mxan7204* cloned into between EcoRI and HindIII sites; Kan^R^ | This study |
| p452_mxan7204_H11A | p452_7204 derivative with H11A mutation in *mxan 7204* gene; Kan^R^ | This study |
| pET28a(+) | Expression vector containing an N-terminally 6xHis-tag | Novagen |
| pET28a_mxan0171 | pET28a(+) with *mxan0171* cloned using NcoI and HindIII sites in frame with C-terminal 6x His tag; Kan^R^ | This study |
| pET28a_mxan0494 | pET28a(+) with *mxan0494* cloned using NcoI and HindIII sites in frame with C-terminal 6x His tag; Kan^R^ | This study |
| pET28a_mxan1555 | pET28a(+) with *mxan1555* cloned using NcoI and HindIII sites in frame with C-terminal 6x His tag; Kan^R^ | This study |
| pET28a_mxan5531 | pET28a(+) with *mxan5531* cloned using NcoI and HindIII sites in frame with N-terminal 6x His tag; Kan^R^ | This study |
| pET28a_mxan7204 | pET28a(+) with *mxan7204* cloned using NcoI and HindIII sites in frame with C-terminal 6x His tag; Kan^R^ | This study |
| pET28a_mxan7204_H11A | pET28a_7204 derivative with H11A mutation in *mxan_7204*; Kan^R^ | This study |
| pET28a_mxan7204_H110A | pET28a_7204 derivative with H110A mutation in *mxan_7204*; Kan^R^ | This study |

**Supplemental Table 3:** Cloning and mutagenesis primers

| **Primers** | **Sequence^a^** | **Description** |
| --- | --- | --- |
| mxan0171F | AAAGAATTCAAAGCCGAGAAAGGTACCGCATGGACGTGATTGACCTGTTGATTCAGCAGC | *G. oxydans* Expression |
| mxan0171R | AAAAAGCTTTCAGGCGTTCGCCTGACCCTGG | *G. oxydans* Expression |
| mxan0494F | AAAGAATTCAAAGCCGAGAAAGGTACCGCATGGAGGTCATCGACCTGTTGATTCAGCAG | *G. oxydans* Expression |
| mxan0494R | AAAAAGCTTTCAGAGGGACGCGCCGCCCCAG | *G. oxydans* Expression |
| mxan1555F | AAAGAATTCAAAGCCGAGAAAGGTACCGCATGGACGCTATCGAACTGTTGGCGC | *G. oxydans* Expression |
| mxan1555R | AAAAAGCTTTCAAATCGGCGCGGCGTGCTCC | *G. oxydans* Expression |
| mxan5531F | AAAGAATTCAAAGCCGAGAAAGGTACCGCATGGATGCATTGGACGTGTTGAACCAGGA | *G. oxydans* Expression |
| mxan5531R | AAAAAGCTTCTAGAACGAGCACACCGGTCCG | *G. oxydans* Expression and Protein Expression |
| mxan7204F | AAAGAATTCAAAGCCGAGAAAGGTACCGCATGGATGCCATCGCGTTGCTGAAGG | *G. oxydans* Expression |
| mxan7204R | AAAAAGCTTTCACGGACCGCTGGCTTCGGCG | *G. oxydans* Expression |
| mxan7402F | AAAGAATTCAAAGCCGAGAAAGGTACCGCATGTCAGCTCGCAGGCATACTCAGG | *G. oxydans* Expression |
| mxan7402R | AAAAAGCTTCTAGCCGTCCCAATACAGGGAC | *G. oxydans* Expression |
| mxan5531F_H11A | GAACCAGGAGGCGCGCCACATCC | Mutagenesis |
| mxan5531R_H11A | AACACGTCCAATGCATC | Mutagenesis |
| mxan5531F_H126A | GTACCGGGGGGCGACCGCGGTGG | Mutagenesis |
| mxan5531R_H126A | AGGCGCAACCAGTCAT | Mutagenesis |
| mxan7204F_H11A | GAAGGCGGACGCGAAGACGGCGGAG | Mutagenesis |
| mxan7204R_H11A | AGCAACGCGATGGCA | Mutagenesis |
| mxan7204F_H110A | CGTGCGCGCGGCGGTCCTGCAGG | Mutagenesis |
| mxan7204R_H110A | TTCTCCATCAACACCTGC | Mutagenesis |
| mxan0171F_C | AAACCATGGGCGACGTGATTGACCTGTTGATTCAGCAGC | Protein Production |
| mxan0171R_C | AAAAAGCTTGGCGTTCGCCTGACCCT | Protein Production |
| mxan0494F_C | AAACCATGGGCGAGGTCATCGACCTGTTGATTCAGCAG | Protein Production |
| mxan0494R_C | AAAAAGCTTGAGGGACGCGCCGC | Protein Production |
| mxan1555F_C | AAACCATGGGCGACGCTATCGAACTGTTGGCGCA | Protein Production |
| mxan1555R_C | AAAAAGCTTAATCGGCGCGGCGTG | Protein Production |
| mxan5531F_N | AAACATATGGATGCATTGGACGTGTTGAACCAGGA | Protein Production |
| mxan7204F_C | AAACCATGGGCGATGCCATCGCGTTGCTGAAGG | Protein Production |
| mxan7204R_C | AAAAAGCTTCGGACCGCTGGCTTCGG | Protein Production |
| mxan7402F_C | AAACCATGGGCTCAGCTCGCAGGCATACTCAGG | Protein Production |
| mxan7402R_C | AAAAAGCTTGCCGTCCCAATACAGGGACTCC | Protein Production |

^a^Restriction endonuclease recognition site is underlined.
